# Stories of women's marriage and fertility experiences: Qualitative research on urban and rural cases in Bali, Indonesia

**DOI:** 10.12688/gatesopenres.14781.1

**Published:** 2023-10-11

**Authors:** Anastasia Septya Titisari, Luh Kadek Ratih Swandewi, Carol Warren, Anja Reid

**Affiliations:** 1 Research Center for Population, National Research and Innovation Agency Republic of Indonesia, Central Jakarta, Jakarta, 10340, Indonesia; 2 Department of Training and Development, National Population and Family Planning Board of Indonesia Bali Representative, Denpasar, Bali, 80234, Indonesia; 3 Global Studies, Murdoch University, Murdoch, Western Australia, Australia

**Keywords:** culture, family, fertility, gender, marriage, population policy, qualitative, social system

## Abstract

**Background:** As a Hindu-majority province in Indonesia, Bali presents a unique and distinctive culture. Patrilocal ( *purusa*) marriage and patrilineal inheritance as a continuation of the patriarchal system puts a man in the key role as a family successor. Having a son is a priority for a married couple in Balinese society. As a consequence, Balinese women experience several constraints related to their economic productive, reproductive, and *adat* (ritual) roles. When a family does not have a male heir, their daughter is pressed to find a spouse willing to accept *sentana* (matrilocal) marriage. This secondary form of marriage brings another complication for Balinese-Hindu women and does not necessarily relieve their submissive position.

This research analyzes Balinese-Hindu women’s perspectives on their marriage experiences and fertility decisions. **Methods:**The data was collected in two areas representing rural ( *Banjar* Tumbakasa in Gianyar) and urban ( *Banjar* Biaung in Denpasar) locations in Bali Province, Indonesia from November 2019 to February 2020. Primary data was based on in-depth interviews of six rural and six urban married Balinese-Hindu women.

**Results:** This qualitative inquiry into Balinese women's experience of the marriage system and fertility options in urban and rural Bali revealed varying degrees of social expectation to provide male descendants for their families. At the same time, economic burdens still haunt them in this development era, and have conflicting implications for family size. Their stories of *purusa* (patrilocal) and *sentana* (matrilocal) marriage were complex, being strongly associated with customary law ( *adat*) in *traditional* society. Paradoxically, however, it was rural women in the study sample who disproportionately opted for the *sentana* arrangement and limitation of family size.

**Conclusions:** This study explores women's fertility aspirations, notably regarding son precedence. It problematizes the *sentana* marriage alternative as a solution to lighten the expectations and burdens affecting women.

## Introduction

Patriarchy places preponderant power and resources in the male sphere, putting women in subordinate positions ^1^. "Patrilocal residence" ^1^ and "patrilineal descent" ^2^ are widely associated with the domination of women in the kinship sphere ^2^. Marrying into a patrilocal (and patrilineal) household creates subordinating circumstances for a woman because, as a guest, she has to obey her husband and his family's authority ^1^. Bali has a patriarchal social structure in which patrilocal and patrilineal systems influence the inheritance pattern, where the heir of the family's property is passed through the male line (son) ^3^.

In Balinese Hindu patrilineal culture, inheritance, political, and household representation are vested in the male line ( *purusa*). Under the patrilineal features of Balinese *adat* (customary) law, a son will inherit family properties and religious responsibilities in the *sanggah* (family temple) and fulfill obligatory community services ( *ayahan*) in the hamlet ( *banjar*) and traditional village ( *desa*) ^4, 5^. On the other hand, Balinese-Hindu families with no son as an heir have alternative options to continue the family line ^6^.

Balinese culture provides alternative pathways to families with no male offspring. The family line and ancestral connection can continue by adopting male offspring from other families of the same clan ( *soroh, dadia*) or arranging " *sentana rajeg*" marriage for a daughter, reversing the usual pattern of female exogamy. *Sentana rajeg* consists of *sentana* (offspring, descendant, successor) and *rajeg* (strong, sturdy), which means that a daughter, in *adat* status, may be confirmed as *purusa* or family successor ^5^ in the absence of a son. In the Hindu law literature, *Manawa Dharmasastra* IX: 127 ^6^ (762), through *sentana rajeg,* a daughter's *adat* status may be transformed to that equivalent to a son ( *putrika*) as family successor. For several Balinese communities, *sentana* marriage is a "solution" in terms of staving off a break in generation, which has been regulated through customary law (awig-awig) ^3^ ^7^.

Thus, although a preference for sons is the normative family form, the patrilocal pattern is modified by the availability of an "alternative" *sentana* marriage through daughters in Balinese society (Adnyani 2017). This marriage is a relatively common, if not preferred, "solution" to the structural demands for a male heir in Balinese culture. Nevertheless, Balinese women are inclined to add more children to their families until they have a son, even though they may themselves do " *sentana*" marriage ^8^. Balinese women experience a high level of expectation from their family and society, especially in their reproductive role, to have a son. The existence of alternative pathways does not necessarily reduce reproductive role anxieties for Balinese women ^5, 12, 13^.

Balinese women face the dilemma of maintaining their identity and vital role in this rapidly changing society. Their main tasks are perceived to revolve around becoming good women, good mothers, and good wives to attain good *karma* for themselves and their descendants ^14^. Their challenge is to negotiate between modern globalizing society and the core values of being Balinese-Hindu. Balinese women carry a "triple burden" due to productive, reproductive, and ritual obligations ^4^. Their roles in the family's economic development, their reproductive role (child-bearing and nursing), and their customary ( *adat)* duties compound the pressures they experience in society ^15^. Indonesian government policies on family planning ^5^ and local concern with cultural preservation significantly influences their marriage and fertility decisions.

This research presents the voices of Balinese women regarding their fertility decisions and the *sentana* alternative marriage option. The results of this study aim to offer new understandings for policy-makers, practitioners, and society in general concerning women's attitudes toward their fertility rights and their roles in society, in particular, related to expectations of their contribution to family and community well-being.

## Methods

The data for this paper is based primarily on 12 in-depth interviews collected in rural (Banjar Tumbakasa in Gianyar) and urban (Banjar Biaung, Denpasar) locations in Bali from November 2019 through February 2020 as part of a thesis research project. ^6^ The following criteria for community respondents in Banjar Biaung and Tumbakasa were applied: six women in each site were chosen who are culturally Balinese-Hindu, aged over eighteen years old, married with at least one child, and gave consent to be interviewed. Respondents were selected through a simple random sampling using the Random Number Generator (RNG) application in two hamlets chosen to represent urban and rural socio-economic contexts. The particular hamlets selected had previously been Family Planning Authority (BKKBN)'s survey research subjects for a Family Data collection program ( *Pendataan Keluarga)* ^16^, so the data obtained from in-depth interviews could be placed in a broader demographic context. A copy of the interview guide used can be found under *Extended data*. ^17^

 Researchers used data triangulation with in-depth interviews, document analysis, and observation to enhance the validity of the research. The interviews were transcribed, coded, and interpreted to examine their attitudes and experiences of marriage and fertility decision-making. The interview questions concerned their fertility decision experiences as Balinese women under the patrilineal *purusa* and alternative *sentana* marriage patterns. The following identification system consisting of a combination of letters and numbers indicating rural or urban residence is used throughout the article to represent each respondent. For example, "Kota01" refers to urban (" *kota*") respondent number 1. Rural participant number one is indicated by "Desa01" ( *desa* = village).

The document analysis included studying local government regulations, documents on Balinese customary law, and publications related to women's position in this context. With respect to observation, the researchers did not have sufficient opportunity for close engagement with the participants beyond the interview context because of the time limitation on fieldwork and participants' busy lives — related to the approaching *Galungan* and *Kuningan* festivals. Therefore, the participant observation component was necessarily limited.

Through qualitative inquiry, this study conveys Balinese-Hindu women's voices about their decision-making regarding marriage and reproduction. The study explores the interview participant's marriage choices and fertility experiences.

### Ethical considerations

Ethical approval was obtained from the Murdoch University Human Research Ethics Commitee (project number 2019/177). Prior to interviews, individual consent was obtained for participation and for the session to be audio-recorded. The information letter provided the following privacy and confidentiality protocols: notes and recordings are to be used only by the research team to ensure correctness of the data and are not available to any other person. Furthermore, the responses are confidential and participants’ names are not used in publications. The data are anonymised and can no longer be directly linked to an individual.

### Respondent characteristics

The 12 women participants in this study comprise six rural and six urban Balinese residents, ranging from 30 to 49 years old. Most Balinese rural participants had secondary education or lower, while the urban resident participants included an equal number of tertiary and secondary or lower graduates. Respondents in both groups mainly worked at home or in the informal sector as retailers, craftswomen, homemakers, and casual farmers. See Table 1.

**Table 1.** Respondents' characteristics.

Variables	Urban (n=6)		Rural (n=6)		Total (N=12)	
	n	%	n	%	N	%
**Age**						
30–39	2	33.33	1	16.67	3	25
40–49	4	66.67	5	83.33	9	75
**Education**						
Secondary education or lower	3	50	5	83.33	8	66.67
Tertiary education	3	50	1	16.67	4	33.33
**Occupation**						
Working at home or informal sector	3	50	5	83.33	8	66.67
Formal employee	3	50	1	16.67	4	33.33
**Number of children**						
0	0	0	0	0	0	0
1	1	16.67	0	0	1	8.33
2	1	16.67	5	83.33	6	50
3	4	66.67	1	16.67	5	41.67
4	0	0	0	0	0	0
**Have son(s)**						
Yes	4	66.67	3	50	7	58.33
No	2	33.33	3	50	5	41.67
**Balinese marriage status**						
Patrilineal ( *purusa*)	5	83.33	2	33.33	7	58.33
*Sentana*	1	16.67	4	66.67	5	41.67

**Source: first author's project survey data**

Five of twelve participants from urban and rural Bali cohorts have three children ( Table 1). Against expectation, the rural women in the sample had fewer children than urban women. Four of six (66.7%) Balinese urban participants have more than two children, while only one of six (16.67%) Balinese rural participants have more than two. Correspondingly, the data update in the government *Pendataan Keluarga* or Family Survey 2015 ^16^, accessed on 12 December 2020, shows that only 23.53% of couples of child-bearing age in the rural area of *Banjar* Tumbakasa have more than two children. Meanwhile, 27.76% of the urban Biaung couples of child-bearing age have more than two children ^16^, see Table 1.

Based on information from the Tumbekasa village chief, the proportion of households married *sentana* in that village is 10/32 households. The Biaung chief confirmed that five households married *sentana* (from 502 households) in his urban *banjar*. Five of the twelve participants have only daughter(s) in this patrilineal culture ( Table 1), which formally preferences the male line. Among 12 participants, five women have married *sentana,* most among the rural participant cohort. *Sentana* marriage confers the status of inheriting male on the daughter and adopts an in-marrying male as the symbolic "wife". However, in practice, the in-marrying husband typically carries out male duties in village social and ritual activities ^5^.

## Results

The form of marriage critically influences Balinese women's legal status because it determines rights and responsibilities in the family and the broader customary community ( *banjar/ desa adat*) ^5^. Most Balinese women participants feel the strain from the system arising from patrilineal marriage, especially the need for an heir, which tends to translate male precedence into son preference. The following discussion covers two themes. The first section includes rural and urban Balinese participants' stories of their experiences in the standard *purusa* marriage system and the importance of having a male heir. The second section shares their experiences and opinions about normative female exogamy, son preference, and the ubiquitous but less valued *sentana* option by which a daughter is symbolically taken as heir to continue the family line, with her spouse marrying into the household.

### a. Stories of *purusa* marriage

*Purusa* marriage refers to the male line through which the responsibility to maintain and carry out ritual ceremonies at ancestral shrines ( *sanggah*) and customary obligations ( *ayahan*) to the banjar community and *adat* village are inherited. It defines the structural power of the male position in the Balinese patrilineal family system, especially concerning inheritance and social/ritual obligations ^7^ ^20^. Seven of twelve Balinese women from the two study cohorts shared their experiences of their position in the household and society in this patrilineal system. These stories reflect various experiences related to this standard marriage pattern, with implications for family planning, fertility decisions, and women's role in society.

***a.1 Urban women's *purusa* marriage and fertility experiences.*** *Purusa* marriage is prominent in Denpasar (an urban area in Bali Province). In the study results, five of six urban women respondents had a *purusa* marriage. The following quotes from the in-depth interviews delve into their marriage experience and implications for fertility decisions.

**Kota01 (46 years old, urban Balinese, a small retailer, mother of two daughters and a son)**
  Indeed, in Balinese society, [women] should have a son. If they don't have a son, they will always try, and it can mean having five kids. Commonly, they will stop if they already have too many kids. Maybe they could ask for a son. *Nyentana.* The man has to get married and live with the women's family... There is a stigma of being a *sentana.* So, many men don't want to do sentana, even if they have economic limitations [in their household of birth].
I agree with the national program of two-children. I do not have two, but three because Balinese must have a son for the family line. A girl will get married and leave; a son will stay at home. *Purusa* is the [customary] *adat* system of succession. I have three children, and the last is a boy. The first two children are girls.
... after the second child, I got pregnant with a boy. Then, I stopped [having more children] and used contraception. Having three children was not my intention. Maybe if I had a boy and a girl, I would have stopped. My husband wants one more because he likes babies. *Aduh* [voice of distress] no more! It's enough. Taking care of a baby means I cannot go to work, or to socialize. I cannot go to the *banjar*, only stay at home. My husband accepts this [the decision not to have more children], and I never take out my IUD. It's my choice [to use contraception]. I won't have more kids. I have two girls and a boy - Complete!

In her account, Kota01 describes common Balinese fertility attitudes and values revolving around the intense social pressure under the patrilineal system for having a son. The couple's fertility decisions were strongly influenced, on the one hand, by the pressure to have a male heir and, on the other, by her desire to limit the number of children through family planning. After having a son, the participant asserted her autonomy and, despite her husband's preference for another child, chose to stop having more children and to use contraception.

**Kota03 (36 years old, urban Balinese, office worker, mother of a daughter and two sons).**
I have three children…. Actually, I wanted two kids, but Balinese have *sanggah gede* [core family temples with major ceremonies]. Only my parents [in-law], husband, and brother-in-law [are responsible]. So, we needed to have heirs. Three kids are enough for me because of the economic burden first and then *adat*…All of it needs money….
I had stress when I got pregnant with my first child. Moreover, my husband [always said] "boy" "boy". Not other people, my own husband. He asked, "Is it a boy, doc?" Then the doctor lectured my husband, "You should be grateful for having a kid when others can't." After that, my husband said, "If it is a girl, we can try again." Yes, a boy is expected because he will stay at home. He will be responsible for the house. …..
My in-laws want to have two boys. Who am I?..... My husband always asks [for more kids] to make the house more lively. But, the cost and the one who gets pregnant is me... "Do you want to get pregnant?" I said to him. It doesn't matter if I only have daughters. Whatever. I don't want more kids.
It is not the mother's fault [for only having daughters]... Yes, women are always blamed... I have a friend who has all-female siblings. What else can they do?... All get married, they leave [the parents]. Sometimes, they will come to visit. They did not marry *sentana*. Only one daughter stays with the parent until they pass away. After both parents have died, then [the house] will be handed over to the extended family.
A woman should be strong to be able to make decisions. [Wishing for] equality, most women take on more jobs, at home, at the office, for *adat,* but still have to respect their husbands. He is the decision-maker, but there should be teamwork. This is not only a woman's job. Men should help. I think it is important to nourish that concept in a family environment. I want my boys to be able to prepare offerings ( *nanding canang*) one of the women's customary roles. I teach them, even though they are boys. Sometimes they refuse because it is the woman's job. I like it when men can help with the "women's job". Because women are prohibited from making offerings *(banten*) during their menstrual periods, so the boys can help.

Kota03's statement also reveals the pervasive importance of the male line in Balinese culture and the intense pressure to produce a male heir from her husband and in-laws, rationalized by ancestral inheritance and the male head of household norms. In this story, the participant expressed resentment of the patrilineal burden and pushed to modify aspects of the customary division of labor in socializing her children.

**Kota04 (41 years old, urban Balinese, civil servant, mother of a son).**
I think having two children is ideal, not too troublesome. Moreover, I am working. Having four children is good for nurturing Balinese tradition, but as I said, it depends on financial ability..... It is a pity for the kids if we cannot afford [their needs]. Thank God, I have a boy. I can be more relaxed. Balinese society prefers a son. Even if there is only one child, I still feel more relieved than if I had a girl. However, both are equal. It's still uncertain that boys can take good care of their parents. Still [a boy is always desired] because he is the family successor. We can not move the *sanggah* [house temple]..... The daughter should leave after marriage. In some cases, women will ask their boyfriends to marry *sentana*. They will change their positions so that the woman is the head of the family...
... I want to help [by working] because I see my husband only has a moderate economic status, and I have skills to work. My husband also supported me, even after I had a child. There was a time when I didn't have anyone to care for my little one. My sister-in-law also said, "Don't quit. What can you use to buy milk? Everything is expensive. We will work out who can take care of him". Sometimes, I took him to my office, which was all in the past. No one [protested] because my co-workers are all women in the same boat.

Like Kota03, Kota04 also views economic difficulty as a strong consideration in reproductive decisions. She argues that a son or daughter has equivalent value. Nonetheless, the community still privileges a boy for family continuity while recognizing *sentana* marriage as a solution for those with no male heir. Somewhat compensating for the inequality of the normative Balinese marriage pattern is the support from her husband, extended family, and colleagues in recognizing this triple burden, especially in resolving the demands of professional and parenting roles.

**Kota05 (46 years old, urban Balinese, an assistant at a nursing home, mother of two daughters and a son).**
Previously, I only wanted to have two children because, under Suharto [New Order era], KB [family planning acronym] was two. But at that time, I didn't have a son. Then at 38–39 years old, I had a boy. .... Absolutely, I would feel distressed if I didn't have a son. The pressure was from myself, as well as from my family. My husband has a nephew, but having my own son is different. Every day, I prayed for a son, so my family could have heirs for our legacy– a cultural legacy. If I were still younger, I would want more [children]. In the Balinese Hindu religion, having more than one boy can lighten up [by sharing] their obligation to take care of their parents, especially for *ngaben* [Balinese cremation] and the series of post-creation ceremonies. It is too hard for only one son.
... I am grateful. Although there is a lot of preparation for *adat* ceremonies, I don't feel tired. It is compulsory to pray.... but there is no burden. If we enjoy the process, we will never feel tired... I can manage it all... I would be more stressed if I didn't have a son.
Balinese have a big responsibility with *adat* ceremonials, such as *ngaben*. ..... Even though we don't have any land or any amount of inheritance, boys are required for the family temple [ *sanggah].* When we have a load of *adat* work to do, the bits of help from others can make my day. For example, my husband will help me when I have a lot of *jaritan janur* [coconut leaf offerings to be prepared for *adat* ceremonies]. I feel happy. So, Balinese women don't feel alone.

Kota05 also expresses the social pressures that led her to have a third child. She was well aware of Indonesia's two-child policy campaign for economic well-being. However, her need to bear a son was absolute. Her fertility preferences were not based on an ideal number of children, but rather the importance of a male child to support household ritual obligations. In her case, the cultural pressures placed on Balinese women produced a double-sided consciousness of the heavy load of *adat* tradition on the one hand and the contentment that came from the collective satisfaction of those obligations.

**Kota06 (37 years old, urban Balinese, a school administrator, mother of three daughters)**
From the government's point of view, the KB program is promoted for population control, but I never use KB [contraception]. So, I do not understand much about it. ... The wish [to have a son] is still there because of Balinese *adat,* [we must preserve] tradition into the future. I wish [to have a son], but back to the economic problem [of having more children]. So I am in the middle of yes or no.
There was no pressure from family because this era was not as traditional as it used to be, especially now that people can think ahead. So, we are not too [fanatical]... No, no. It is only a desire, but it does not make me depressed that I should have a boy..... *Astungkara* [thank God], so far, in-laws or parents have never asked me to have a son or try again.
Yes, my husband still expresses his worries about the future if we only have daughters, "Who will take care of us?" Ah, now the era is different... *Nyentana.* Asking for the boy to come to the girl's home. With *sentana*, the system is better. If the boys are reticent, we will personally invite them to stay with us. ... but they will still do the banjar *adat* and civil obligation at the boy's own family's house. ... a double *banjar* obligation. ^8^

Kota06 expresses considerable ambivalence in describing her fertility decisions. She does not use contraceptives even after having three children due to the high value of a son in the Balinese community. Aside from *adat* obligations, support in old age is a crucial consideration arising from patrilineal arrangements. While *sentana* marriage for one of their daughters would resolve this dilemma, there is a normative patrilineal model that weighs against an equal valuation of the sentana option. She also considers a new form of marriage *pada gelahang* that has evolved as a potential means to override her own and her family's burdens. The stories above show that even urban women who have careers outside the home felt pressure to have sons, and at least four out of five who have *purusa* marriages had an additional child to ensure there is an inheriting son to fulfill their task as Balinese wives. Three participants said they did so explicitly to satisfy patrilineal inheritance norms. It is undeniable in the Balinese context, that normative social expectations of women's roles shape and influence their fertility choices and reproductive agency. In this social construction, "women's destiny" was commonly idealized as a measure of the quality of womanhood. This is the case in Bali as well as across Indonesia ^21^. 

***a.2 Rural women, family planning and purusa marriage.*** The conventional description of a woman's place in Balinese social structure is expressed in the following rural woman's perspective on marriage:

**Desa01 (47 years old, Balinese villager, a homemaker and casual farmer, mother of two daughters).**
I would happily have more kids if they have a [guaranteed] future. If they don't have it, I won't. I am not going to be forced to have more children... two are enough ... I can not provide enough food for many kids. If other people have more (money), they will have four ^9^ For me, two are enough. It is okay... more importantly, I can take care of my kids within my limitations….
At 18 years old, she [one of the participants' two daughters] got married by *ngidih sentana* [seeking *sentana]*. *Ngidih sentana* is difficult. I don't know about [how she did it]. After four days, they got married. ..... I feel grateful to have gotten a *sentana* even if I don't have [assets]. I am really grateful. I have two grandchildren [a boy and a girl]. Safe. It was hard to find *sentana* because fewer [men] would marry outside... [Even] if [the parents] have three sons, they will prefer to stay at home. It was hard..... He [the son-in-law] is from Kintamani, Bangli. He consented to a *sentana* marriage because he had five siblings, three of them are boys who had only a single cramped house.

As a casual farmer and homemaker, Desa01 explains that economic limitations outweigh her fertility preference to have more children. She stopped after two pregnancies, even though she had no son. Not only did economic constraints influence her fertility decisions, but analogous economic difficulty by her account was a driving factor of her son-in-law's agreement to a " *sentana*" marriage with her daughter.

**Desa04 (41 years old, Balinese villager, homemaker and casual farmer, mother of a daughter and a son).**
It is better to have two [children]. I already have a boy and a girl. I am grateful. I wanted that, and I got it. I use the IUD. It fits...... and is safe. No [side effects or complaints]. I feel comfortable.
The pressure to have a son is still intense in my community. It will always be problematic. Having two daughters is a problem. Having *sentana* is still a problem. Then, telling the daughter to make a lot of boyfriends that could be a [marriage] candidate always becomes a problem. After she grows up, the daughter still gets blamed.... the parents complain, and the daughters are more upset. It's a pity.
Indeed, [Balinese women] obey their husbands and are submissive. I don't want my husband to follow me. I don't want to. I was born and aware of my situation as a woman.
No, my father-in-law will be a decision-maker. Then, my husband. Because I still have a father-in-law, my husband will obey his father's decision. Here, parents come first... No, I am a follower. My in-law and my husband had better make the decisions. I don't want to get involved. I think I am not able, incompetent. It is better that I obey.
Because I stay with my husband's family, he carries more weight. It would be different when the husband lives with the wife's family. Also, I no longer have rights at my family's house because I got married outside. I only give them a visit…. I am a woman. It is a common thing for Balinese.
When we have many *adat* ceremonies *....* sometimes we [the participant and her women friends] complain. Even though it is a Balinese routine, I usually share my weariness with my friends. When we share and talk in the *banjar*, I feel more relieved.

Desa 04 shares her experiences as a rural Balinese woman who left her natal family for the normative *purusa* marriage. She eventually exercised fertility autonomy in her decision to use contraception after fulfilling her obligation as a wife to bear a male heir for her husband's family. Her story portrays a typical rural Balinese woman as a victim of the patrilineal system in society, normative values she has primarily internalized. Nonetheless, the women's sphere also offers a support network that accompanies the intense social interaction in ritual and community work. Exchanging stories in the *banjar* makes her realize that she is not alone.

### b. Stories about *sentana* marriage

In Balinese society, typically, male descendants have rights to family inheritance. In an alternative marriage system partially parallel to female exogamy, men who marry *sentana* lose these rights. Men no longer have formal rights or responsibilities after they leave their natal family ^20^. Moreover, *sentana* marriage is a step taken to maintain the family line even though the family only has daughters. This marriage aligns with the national family planning program focusing on small, happy, and prosperous families benefiting from Balinese society's development ^4, 5^.

The Balinese community's acceptance of this marriage form varies and depends on each region's *adat*. Tabanan and Gianyar regions accept and have practiced *sentana* marriage from generation to generation ^5^. Other areas of Bali province, such as Jembrana, Karangasem, and Klungkung, have not recognized *sentana* marriage ^5^. This uxorilocal marriage has weak legal status and misses the three legal components of law: legal structure, legal substance, and legal sanctions ^5, 7, 24^. S *entana* marriage is an *adat* (customary) law practice, not a rule of Hinduism ^5^. This marriage depends on the local provision of *awig-awig* or Balinese customary law, which may differ from one *adat* village to another in Bali ^7^. Five women were in a sentana marriage relationship among the twelve rural and urban interview participants. However, interestingly only one of these was an urban participant, which might have been expected because *sentana* marriage is not common in the Denpasar community ^5^.

**Kota02 (45 years old, urban Balinese, *sentana marriage*, homemaker and online retailer, mother of two daughters).**
In Balinese culture, women marry outside. It is an obligation. Because my brother passed away, I had to stay at home. I asked [my husband] to stay with me because of that situation. Yes, *nyentana...* I cannot marry outside because there are no heirs. I have cousins, but the one who has rights here is me. My husband agreed to do it [ *sentana*]... He bears a heavier burden because he salso had to change his faith ^10^. His family agreed. It is also what he wants. I am not pushing him. If I force him, I'm afraid it will be a problem in the future. Commonly, after *sentana* and having children, the husband leaves.
If I had two sons, two would be enough. Because I have two girls, I want one more child. If it is not my luck, it won't matter. Enough. Girls are the same [as boys]. [I'm] still grateful. One of them can stay at home, like me. I told my husband we would always be grateful if we only had two girls.

In the experience of Kota02 the pressure did not stop after she found a partner willing to marry *sentana* but continued after her two pregnancies. She feels that she needs to have a son, or one of her daughters will repeat her experience because of the strength of the patrilineal system in the Balinese community. In her *sentana* marriage, Kota02 recalls that her husband also experienced pressures due to their marriage, which points to other perspectives on the experience of *sentana* for men who can also be 'victims' of cultural values and socio-economic structures.

**Desa02 (47 years old, Balinese villager, *sentana marriage*, a homemaker, mother of two sons).**
It is easier for a man to find a woman but harder for a woman to ask a man to stay at her home. Balinese men have a different status. ... A woman will not be[come] a man [through *sentana*]. The head of the household is still the husband, not me. The difference is that the inheritance is the woman's treasure.
I am one of two sisters. One left the house. I stayed, and sought *sentana.* I lived and was born here. I found a husband [a neighbor]. I had [two] sons. A neighbor took one for " *sentana*". ... No one leaves their parents here. ... Together, they care for their parents... It is not a big deal for me [because] my [other] son, his wife, and grandchildren often visit.

Desa02 reflects on her fertility decisions as a rural woman and homemaker in the context of her own *sentana* marriage and that of one of her sons. She is grateful that she has two sons and that the one who lives with his wife is close enough to visit his mother often. A mitigating factor in both types of marriage is the solid traditional preference for in- *banjar* marriage, which means that close relationships are usually maintained regardless of the marriage pattern.

**Desa03 (43 years old, Balinese villager, *sentana marriage*, a homemaker and craftswoman, mother of two daughters).**
I agree [with the two-child program] because taking care of two kids is easier. More children, more stress. I have two girls. No, no, no. I don't want to have more. Two are enough. It is easier. The issue is economic. I only have cramped land. Where will they stay? Rich people have more children. I accept my fate.
I was *ngidih* [proposing *sentana*] because I was the one and only heir of my family. It was hard [to persuade the prospective husband to *nyentana*]. Today's era is even more difficult. In the past, my parents took care of it. They did not introduce him to me, but our marriage was an arrangement. Now, children won't do that. She will look for [a husband] by herself. I was stupid. I can't do things right... more important to have a descendant.
I want to have a son..... but what if I have more kids, and it is a girl... I lay my faith in God. [one of the participant's daughters is prepared to marry *sentana*].
My husband is still the head of the family. Even though he is from the outside, he is still my husband. [the husband] has equivalent rights as a son. He is still my husband, a man. Always, because he is the man and I am only a woman..... As a woman, I don't dare to disturb my husband, asking for this and that. I don't dare. Let him be. Yes, my husband is still 'number one'.

The pressure on Balinese women does not stop after finding a *sentana* candidate and marriage. Male heirs are still critical for *adat* continuity in the next generation. Desa03 experienced a difficult and rocky journey as a daughter with no brother, the wife of *sentana* husband, and a mother with no son. Social pressures were multiplied by economic limitations as a rural homemaker and craftswoman. For her, the economic factor ultimately outweighs any advantage in producing a male heir.

**Desa05 (31 years old, Balinese villager,** *sentana marriage* **, an honorary teacher, mother of two daughters).**
Two children would be enough if I had a son and a daughter. Sadly, I have two girls. Yes, later [maybe another baby]. The pressure is actually from me. I really want [a son]. I'll be grateful if I have one, but it'll be okay if I don't. More importantly, I have children rather than nothing...
Balinese men prefer to stay at their own home, so asking them to stay at a women's place will be hard. Rarely will the men obey. ... Maybe, it's more comfortable to stay. Moreover, finding several brothers who will remain at their family's home is essential. That's the difficulty.
*Aduh,* Balinese have a lot [ *adat* activities]. I have been performing *adat* work ( *ngayah*— Balinese mutual cooperation) for one week at the temple *.* Then, two days later, I will have a ceremony at my house. I am still fortunate to have a mother and aunt who always stay with me and can help [thanks to her *sentana* marriage], so I can go to work. In this *banjar*, mutual help among neighbors is a tradition.

In concert with Desa03, Desa05 shares her story on population policy versus patrilineal pressure. She also experiences Balinese women's triple burden, which is productive (working as a teacher), reproductive (bearing male heirs), and customary (ritual *adat* activities). However, the participant gains support from other women and neighbors to alleviate her triple burden as a rural Balinese woman.

**Desa06 (40 years old, Balinese villager,** *sentana marriage* **, a small retailer** *,* **mother of three sons).**
Men are expected to be the heirs of the family. It is the principle. If the first baby is a boy, the second will be easier; whether a boy or girl doesn't matter. If the first child is a girl, the second will be more difficult. Looking for sentana is harder. Yes, it was not easy when I was looking for [ *sentana*] here [neighborhood]. I had to go looking far afield. .... Men rarely want to look for sentana. Men think it is better to stay at home. Doing *sentana*, men live in someone else's family...
I was *ngidih* (looking for) a *sentana*... I have two siblings, all girls. Now, I have three boys. ... I should obey my husband to keep him calm—making him comfortable, following him. I should control my speech so that he won't get offended. I will comply with my husband's decisions and not cause him distress; make him feel at home. .... I am indeed the heir, but he is responsible for the kids' tuition fees. As a woman, how much do I earn?
The husband's role is more important than mine as a woman. He is the leading actor and takes responsibility for everything, including *adat*. I feel sorry for him when there are more demands, like the children's tuition fees. He gets a burden, but I cannot help. Sometimes, I feel sorry.
Suppose other families ask for a son [because I have three]. If my son wants to, I will give him to them. Yes, I experienced the difficulty of looking for a *sentana.* [But] I also should be ready for all my kids if they choose to stay at home after marriage.

Even though she sought *sentana* and has the right to her family inheritance, Desa06 still feels inferior to her husband. She feels the need to compensate for his agreement to enter a *sentana* marriage. Desa06's story reflects a process of learning from the " *ngidih sentana"* marriage experience, which contributes to her personal openness to a son following this path, out of sympathy for both the needs of other women in this position as well as the personal marriage choices of her children.

### Discussion/Conclusion

The strong patriarchal norm in Indonesia contributes to women's social problems ^25^, especially Balinese-Hindu women. The patriarchal system arises as the center of Balinese society's social structure through patrilocal and patrilineal religious and customary traditions ^26^. The system pressures married Balinese-Hindu women to have children, especially a son as an heir, and to continue the husband's family's lineage ^26^. The emphasis on having a son causes some women to add to the number of children they have with the hope that the next child will be a boy ^27^. The interviews with rural and urban women reveal that most participants experience the pressure to have a son in their marriage as a burden.

 The Balinese patrilineal inheritance system places the male at the center of the social structure ^20^. Male descendants are considered the main actors who carry the inheritance, name, and responsibility for the family, making them highly valued in the community. Balinese women participants reveal their fertility attitudes in the context of structural pressures favoring patrilineal ( *purusa*) marriage and masculinist values. The variations in women's responses reported above are based on their realistic recognition of social norms and personal life circumstances. Son precedence, if not necessarily preference, is an outcome of those structures and is mainly responsible for the fertility choices and constraints on their perceived options ^13^.

Regarding fertility preference, several respondents in both marriage types decided to use contraception after they gave their husbands a son. The others did not use contraception because they have no son yet. This finding aligns with other studies in Bangladesh where contraceptive use among women with only daughters is lower than that of women with sons (s) because of the influence of the patrilineal system. Consequently, son(s) preference becomes a reason to have a larger family size when they only have daughters ^28^.

Balinese-Hindus believe the ancestral spirit is released from the abyss through the grandson's role ^29^. In Balinese, getting pregnant and giving birth to a child is more than a biological matter. It is connected to culture and religion ^30^. As written in *Rgveda* X.85.42: " *Ihahiva stam ma vi yaustam, visvam ayur vyasnutam Kridantau Putrair naptrbhih, modamanau sve grhe*", meaning: "Yes husband and wife may you stay here and never be separated. Hopefully you both achieve full life happiness. Hopefully you play with your son and your grandsons, and live in this house happily" ^31^. The ancient literature confirms the strain on the family member to maintain lineage continuity. In a *sentana* marriage, the burden of having a son continues into subsequent women's generations in the family, from a mother to her daughter. It is illustrated by Desa01, who chooses not to add another child to the family for serious economic reasons when she has no son. Her decision passes an extra obligation for her daughter to find a man willing to accept “ *sentana*” marriage and give birth to a grandson as an heir to give her family a sense of security.

On the other hand, economic pressure is a structural factor that greatly outweighs son preference in the fertility decisions of these women. This is particularly evident in the rural Balinese women's perspective on *sentana* marriage. The result is in line with a study result in other rural areas in Bali that Balinese women's fertility aspiration are not simply left to God or fate but determined in concert with their husband (and extended family), considering the costs and benefits of child-bearing and childrearing ^13^. Unlike women, the theory that men's status after *sentana* marriage as *predana* or "woman", ^5, 20, 24^ makes their position inferior is not automatic. As portrayed by some respondents' stories, the husband is still the man of the family, and the wife still feels obliged to obey her husband. In one case, a respondent describes feeling the need to serve her husband courteously to honor his "sacrifice" as " *sentana nyeburin*" ^11^. That said, some accounts also describe supportive husbands and extended families.

The findings reveal the multiple roles of Balinese women as wives, mothers, and members of society. The results also show that marriage patterns and fertility decisions face significant cultural and economic drivers. *Sentana* marriage, considered a solution to the prescription for male inheritance, can not guarantee Balinese women's equality. This marriage could be deemed a tool to prevent the loss of the family line and inheritance, and ultimately serving to maintain patriliny. Challenges are raised not only by customary law, but also by constraining economic factors. While not generalizable beyond this small sample, this study, using in-depth interviews, indicates the complexity of internal and external forces affecting Balinese women's fertility decisions. Quantitative and qualitative studies are needed to provide broader perspectives and deeper understanding, especially regarding Balinese women's fertility agency in the context of culture, customary law, and development policies.

## Data Availability

The audio files will not be shared because the interviews may contain sensitive data about personal life and community. However, there is provision for some de-identified transcripts to be provided on request to ensure that use is limited to genuine researchers and to protect respondents’ privacy and the local community. An application from an established researcher to obtain redacted transcripts would require review by the HREC Committee at Murdoch University. A written request outlining 1) the name and status of the researcher, 2) institutional affiliation, and 3) purpose for which the data is sought, would be required of the applicant. Figshare: Stories of women's marriage and fertility experiences :Qualitative research on urban and rural cases in Bali. https://doi.org/10.6084/m9.figshare.23674401.v1 ^17^. This project contains the following extended data: Approval Letter _Balinese story.pdf Oral Information and Consent Form-33512093.pdf Stories of women.docx (interview guide) Data are available under the terms of the Creative Commons Attribution 4.0 International license (CC-BY 4.0).

## Footnotes

^1^ Patrilocal means a marriage pattern where a post-married couple reside with the husband's extended family ^9, 10^.

^2^ Patrilineal means a kinship system based on paternal line ^3^.

^3^ According to Bali Provincial Regulation Number 3 of 2003, *awig –awig* is Balinese customary laws, sourced from *Catur Dresta* and imbued with Balinese Hinduism, with the purpose to manage the society. The *Catur Dresta* consists of *Sastra Dresta* (religious teachings), *Kuna Dresta* (cultural values), *Loka Dresta* (the way of life), and *Desa Dresta* (local customs) ^11^.

^4^ Making offerings and other preparations both for religious ceremonies in the household and in the traditional village ^18^

^5^ Family Planning is one of the most important programs in Indonesian development policy; two children are enough is the family planning program's main slogan, in order to reduce maternal mortality rates during childbirth and control population growth ^19^

^6^ The research was conducted under the Murdoch University Human Research Ethics Committee (project number 2019/177). Because of the requirement for anonymity of respondents, a coding system is used for each respondent.

^7^ In Balinese customary law ( *adat*), male descedants are prefered because they are considered important to preserve their family responsibilities in relation to their ancestors, Hindu religious obligations, and other community and environmental obligations. In traditional Balinese Hindu practice, only males have rights to inheritance (Diatmika and Sujana 2018).

^8^ This is not strictly an obligation under *adat*, but indicates the continued sense of obligation that an out-marrying male child would feel toward his natal family. That said, out-marrying daughters also return to help their families of origin with major ceremonial preparations, though not based on formal obligation. By this double banjar obligation, Kota 06 is referring to a new form of *pada gelahang* marriage, which combines traditional *purusa* marriage and *sentana* marriage. It deems to be a solution when the couple do not want to choose between the two traditional *purusa* or *sentana* marriage alternatives. However, *pada gelahang* marriage is still a controversial concept ^22, 23^.

^9^ Here she alludes to the recent Bali provincial government policy to encourage a four-child family model as a local alternative to the national two-child ideal. See further discussion of the ethno-political rationale for this reversal of national family planning objectives in Titisari *et al*. (2022).

^10^ Kota02’s husband changed his religion because of their marriage, from a Buddhist to Hindu.

^11^ The term for the husband's status in marrying *“sentana”* and following the wife's family line ^32^.
