# Stories of women's marriage and fertility experiences: Qualitative research on urban and rural cases in Bali, Indonesia

**DOI:** 10.12688/gatesopenres.14781.2

**Published:** 2024-10-14

**Authors:** Anastasia Septya Titisari, Luh Kadek Ratih Swandewi, Carol Warren, Anja Reid

**Affiliations:** 1 Research Center for Population, National Research and Innovation Agency Republic of Indonesia, Central Jakarta, Jakarta, 10340, Indonesia; 2 Department of Training and Development, National Population and Family Planning Board of Indonesia Bali Representative, Denpasar, Bali, 80234, Indonesia; 3 Global Studies, Murdoch University, Murdoch, Western Australia, Australia

**Keywords:** culture, family, fertility, gender, marriage, population policy, qualitative, social system

## Abstract

As a Hindu-majority province in Indonesia, Bali presents a unique and distinctive culture. Patrilocal ( *purusa*) marriage and patrilineal inheritance as a continuation of the patriarchal system puts a male in the key role of family representative and successor. Having a son is a priority for a married couple in Balinese society. As a consequence, Balinese women experience several constraints related to their economic productive, reproductive, and *adat* (ritual) roles. When a family does not have a male heir, their daughter is pressed to find a spouse willing to accept *sentana* (daughter succession) marriage. This secondary form of marriage brings another complication for Balinese-Hindu women and does not necessarily relieve their submissive position. This study analyzes Balinese-Hindu women’s perspectives on their marriage experiences and fertility decisions in patrilineal society in changing rural and urban conditions.

The data was collected in two areas representing rural (Gianyar) and urban (Denpasar) locations in Bali Province, Indonesia from November 2019 to February 2020. Primary data was based on in-depth interviews of six rural and six urban married Balinese-Hindu women.

This qualitative inquiry into Balinese women's experience of the marriage system and fertility options in urban and rural Bali revealed varying degrees of social expectation to provide male descendants for their families. At the same time, economic burdens still haunted them in this development era and manifested conflicting implications for family size. Their stories of *purusa* and *sentana* marriage were complex because it has strongly associated with customary law ( *adat*) in traditional society. Paradoxically, this study found that it was predominantly rural women who opted for the *sentana* arrangement and expressed a preference for smaller family sizes.

This study explores women's fertility aspirations, notably regarding son precedence. It problematizes the *sentana* marriage alternative as a potential solution to alleviate the expectations and burdens placed on women.

## Introduction

Traditionally, societies have assigned gender roles based on biological sex, which often favor men with strong masculine traits for leadership roles. The term ‘patriarchy’ refers to both the authority of fathers within families and the broader structural domination of women by men ^1^. In several societies, patriarchy has been influencing fertility variations through gender discrimination and marriage systems, including in Ethiopia, China, India, and Indonesia. In Ethiopia, the intersection between economic development and patriarchy strongly influences women’s lives, including their ability to express their concern for their lives and bodies ^2^. Chinese households traditionally favored males in a patriarchal system, a preference that was exacerbated by the one-child policy in the late 1970s. This policy led to female infanticide and sex-selective abortions, underscoring the persistent influence of cultural norms on demographic trends despite gender equality advances ^3^. In contemporary India, patrilineal and patriarchal practices remain prevalent, especially among Hindu castes, prioritizing male lineage and reinforcing gender inequality ^4^. Despite advances in women’s rights, traditional preferences for sons and male dominance still persist, particularly in Indian rural areas ^5^. Meanwhile, Bali, as one of the regions in Indonesia, provides a unique case related to patriarchal, patrilineal, and patrilocal systems in the context of dramatic economic transformation associated with the development of the tourism industry ^a^.

Bali is considered special because the society adheres to Hinduism in Indonesia, the majority Muslim religion. Balinese Hinduism, with its emphasis on harmony among the divine, humans, and nature ( *Tri Hita Karana*), profoundly influences Balinese life through numerous ceremonies ^6^. On the other hand, globalization has expanded Balinese women’s roles beyond traditional domestic duties to include economic and social responsibilities. They balance family, income-earning, and community activities while maintaining their cultural identity and religious values ^7^. This adaptability allows them to navigate modern complexities while upholding *Tri Hita Karana*, ensuring their contributions to family and community life remain harmonious and balanced.

In Bali, patrilocal marriage and patrilineal inheritance have been seen as a means to preserve Balinese identity, ethnicity, and Hindu religion ^8^. While patriarchy places preponderant power and resources in the male sphere, "patrilocal residence" ^b^ and "patrilineal descent" ^c^ are widely associated with the domination of women in the kinship sphere ^9^. Marrying into a patrilocal (and patrilineal) household creates subordinating circumstances for a woman because, as a guest, she has to obey her husband and his family's authority ^10^. Bali has a patriarchal social structure in which patrilocal and patrilineal systems influence the inheritance pattern, where the heir of the family's property is passed through the male line (son) called *purusa* ^11^. Under the patrilineal features of Balinese *adat* (customary) law, a son will inherit family properties and religious responsibilities in the *sanggah* (family temple) and fulfil obligatory community services ( *ayahan*) in the hamlet ( *banjar*) and traditional village ( *desa*) ^12^. The system places significant pressure on women to produce male heirs.

Balinese culture provides alternative pathways to families with no male offspring. The family line and ancestral connection can continue by adopting male offspring from other families of the same clan ( *soroh, dadia*) or arranging " *sentana rajeg*" marriage for a daughter, reversing the usual pattern of female exogamy. *Sentana rajeg* means that a daughter, in *adat* status, may be confirmed as *purusa* or family descendant ^12^ in the absence of a son. In the Hindu law literature, *Manawa Dharmasastra* IX: 127 ^13^ (762), through *sentana rajeg,* a daughter's *adat* status may be transformed to that equivalent to a son as family successor. *Sentana* marriage involves the woman paying a dowry and the man moving into her family home ^18^. For several Balinese communities, *sentana* marriage is a "solution" in terms of staving off a break in generational succession, which has been regulated through customary law ^18^.

Although it has been considered a long-standing solution for a family with no son, the *sentana* system still marginalizes women by maintaining the patrilineal structure and reinforcing male dominance. The man loses inheritance rights in his original home, while the woman must take a new status to preserve her family’s lineage. The husband’s legal status changes to that of a woman ( *predana*), while the wife’s status remains limited in practice underscoring gender inequality. This practice continues to prioritize the male lineage, often at the expense of women’s autonomy and rights. Balinese women are inclined to add more children to their families until they have a son, even when they have themselves taken on " *sentana*" marriage ^19^. The existence of alternative pathways does not necessarily reduce reproductive role anxieties for Balinese women ^12, 20, 21^.

Furthermore, the interplay between Indonesia’s national two-child policy and the local KB Krama Bali policy profoundly influences women’s reproductive choices ^22^. The national two-child policy, designed to curb population growth, has historically empowered women by granting them greater autonomy over their fertility decisions, enhancing their workforce participation and improving their children’s educational and living standards. Conversely, the KB Krama Bali policy, which advocates four children family ideal to uphold cultural values, complicates these advancements ^23^. This pro-natalist policy emphasizes cultural traditions, such as the Balinese naming system, and heightens the pressure on women to produce male heirs. Consequently, Balinese women must navigate a complex landscape of economic, cultural, and political pressures, often with limited agency ^22^. This tension highlights the necessity for the policies harmonizing cultural values with women’s reproductive rights and economic well-being. Indonesian government policies on family planning ^d^ and local concern with cultural preservation significantly influence their marriage and fertility decisions.

Balinese women face the dilemma of maintaining their identity and vital role in this rapidly changing society ^24^. Their main tasks are perceived to revolve around becoming good women, good mothers, and good wives to attain good *karma* for themselves and their descendants ^6^. Their challenge is to negotiate between modern globalizing society and the core values of being Balinese-Hindu. Balinese women carry a "triple burden" due to productive, reproductive, and ritual obligations ^e^. Balinese women face “triple burdens” as they juggle productive roles like working in the informal sector, reproductive roles involving childbearing and childcare, and ritual responsibilities in religious and cultural ceremonies ^27^. These roles are crucial for their family’s financial stability and maintaining Balinese Hindu traditions. The term “triple burdens” emphasizes the significant challenges and pressures of managing these multiple responsibilities simultaneously.

Understanding kinship structures and gender roles as power relations rather than as a universal force of domination is crucial for a nuanced perspective ^1^. Kinship systems are dynamic entities that continuously evolve in response to social, economic, and political transformations. Analyzing these adaptations provides a deeper understanding of how societies navigate challenges and leverage opportunities and offers valuable insights for managing change in contemporary contexts. In broader contexts, this knowledge also helps create more effective and culturally sensitive policies in areas such as family law, social services, and community development ^2^. This research amplifies the voices of Balinese women regarding their fertility decisions and the *sentana* marriage option, offering valuable insights for policymakers and society. By presenting these perspectives, the study aims to enhance understanding of women’s attitudes toward fertility rights and societal roles, emphasizing the importance of considering women’s voices in shaping policies and practices that affect their lives and communities.

## Methods

The data for this paper is based primarily on 12 in-depth interviews collected in rural (Banjar Tumbakasa in Gianyar) and urban (Banjar Biaung, Denpasar) locations in Bali from November 2019 through February 2020 as part of a thesis research project ^f^. In-depth interviews with a limited range of participants (5 to 25) are regarded as an appropriate ethnographic methodology, particularly where researchers have the background experience and language facility to contextualize and facilitate communication ^28^. *Banjar* Biaung was chosen as a representative urban context because it is located at Kesiman Kertalangu, Denpasar city— the capital of Bali province. Urban areas are considered to reflect more demographic changes because of their role in driving economic change and modernization ^29^. Based on Susenas 2013 and 2016 data, the Total Fertility Rate (TFR) of Denpasar increased from 1.9 to 2.09. According to *Pendataan Keluarga* (Family Enumeration) National Population and Family Planning Board (BKKBN), in 2015, 180 of 245 (73.47%) couples of childbearing ages had two or fewer children. Moreover, the majority of them chose not to have more children (65.31%), and only 27.75% wanted more than two children (BKKBN 2015). In 2014, the percentage of the poor population in Denpasar city was 2.07% (BKKBN Bali 2016).

Located in Tegallalang, Gianyar district, where the majority of the people make their living from agriculture (rice production) and livestock, *Banjar* Tumbakasa has different and somewhat surprising characteristics compared to urban *Banjar* Biaung. The TFR in Gianyar, in Susenas 2013 and 2016, decreased from 2.2 to 2.03. From Family Enumeration data in 2015, 161 of 204 (78.92%) couples of childbearing ages had two or fewer children. Furthermore, 75.98% of them chose not to have more children and only 21.08% wanted more than two children (BKKBN 2015). Around 4.27% of Gianyar's population registered as poor in 2014 (BKKBN Bali 2016).

The following criteria for community respondents in *Banjar* Biaung and Tumbakasa were applied: six women in each site were chosen who are culturally Balinese-Hindu, aged over eighteen years old, married with at least one child, and gave consent to be interviewed. This ethnographic research began with requesting permission from the two *Banjar* chiefs in each location. They were asked to provide a list of members that fit the research criteria. Then, participants were randomly selected from the list using an Android application called Random Number Generator (RNG). The researchers visited their homes to request a meeting and interview. The participants chose the interview appointment date and preferred location. Because the particular hamlets selected had previously been Family Enumeration ^30^, the data obtained from in-depth interviews could be placed in a broader demographic context. A copy of the interview guide used can be found under *Extended data* ^31^.

The researcher used data triangulation with in-depth interviews, document analysis (Family Enumeration 2015 report and Banjar Biaung’s profile), and observation to enhance the credibility of data. The interview data was analyzed by categorizing and labelling the data (coding). The coding process was started by transcribing the interviews verbatim to capture all nuances. Then, we highlighted significant phrases or sentences from the transcripts. We identified several themes, such as fertility experience, *purusa* marriage, and *sentana* marriage. These themes were analyzed by categorizing the fertility experience into *purusa* marriage and *sentana* marriage. The identification system for participants was used to provide anonymity, consisting of a combination of letters and numbers indicating rural or urban residence is used throughout the article to represent each respondent. For example, "Kota01" refers to urban (" *kota*") respondent number 1. Rural participant number one is indicated by "Desa01" ( *desa* = village). With respect to observation, the researchers did not have sufficient opportunity for close engagement with the participants beyond the interview context because of the time limitation on fieldwork and participants' busy lives — related to the approaching *Galungan* and *Kuningan* festivals. Therefore, the researchers’s observation of the participants’ components was necessarily limited. Although the observation data was limited, the researchers used data information from the Banjar’s chiefs to understand the sociodemographic characteristics of each site.

Through qualitative inquiry, this study conveys Balinese-Hindu women's voices about their decision-making regarding marriage and reproduction. The study explores the interview participant's marriage choices and fertility experiences.

### Ethical considerations

Ethical approval was obtained from the Murdoch University Human Research Ethics Committee (project number 2019/177). Prior to the interviews, individual consent was obtained for participation and for the session to be audio-recorded. The information letter provided the following privacy and confidentiality protocols: notes and recordings are to be used only by the research team to ensure the correctness of the data and are not available to any other person. Furthermore, the responses are confidential and participants’ names are not used in publications. The data are anonymized and can no longer be directly linked to an individual.

### Respondent characteristics

The 12 women participants in this study comprise six rural and six urban Balinese residents, ranging from 30 to 49 years old. Most Balinese rural participants had secondary education or lower, while the urban resident participants included an equal number of tertiary and secondary or lower graduates. Rural respondents mainly worked at home or in the informal sector as retailers, craftswomen, homemakers, and casual farmers, while urban respondents’ occupations were equally divided between formal and informal sectors. See Table 1.

**Table 1.** Respondents' characteristics.

Variables	Urban (n=6)		Rural (n=6)		Total (N=12)	
	n	%	n	%	N	%
**Age**						
30–39	2	33.33	1	16.67	3	25
40–49	4	66.67	5	83.33	9	75
**Education**						
Secondary education or lower	3	50	5	83.33	8	66.67
Tertiary education	3	50	1	16.67	4	33.33
**Occupation**						
Working at home or informal sector	3	50	5	83.33	8	66.67
Formal employee	3	50	1	16.67	4	33.33
**Number of children**						
0	0	0	0	0	0	0
1	1	16.67	0	0	1	8.33
2	1	16.67	5	83.33	6	50
3	4	66.67	1	16.67	5	41.67
4	0	0	0	0	0	0
**Have son(s)**						
Yes	4	66.67	3	50	7	58.33
No	2	33.33	3	50	5	41.67
**Balinese marriage status**						
Patrilineal ( *purusa*)	5	83.33	2	33.33	7	58.33
*Sentana*	1	16.67	4	66.67	5	41.67

**Source: first author's project survey data**

Five of twelve participants from urban and rural Bali cohorts had three children ( Table 1). Against expectation, the rural women in the sample had fewer children than urban women. Four of six (66.7%) Balinese urban participants had more than two children, while only one of six (16.67%) Balinese rural participants had more than two. Correspondingly, the data update in the government *Pendataan Keluarga* or Family Enumeration Data 2015 ^30^, accessed on 12 December 2020, showed that only 23.53% of couples of child-bearing age in the rural area of *Banjar* Tumbakasa had more than two children, while 27.76% of the urban Biaung couples of child-bearing age had more than two children ^30^ (see Table 1).

Based on information from the Tumbekasa village chief, the proportion of households that married *sentana* in the village is 10/32 households. Meanwhile, the Biaung chief confirmed that five households married *sentana* (from 502 households) in his urban *banjar*. Five of the twelve participants in this study had only daughter(s) ( Table 1). Among 12 participants, five women had married *sentana,* most among the rural participant cohort. *Sentana* marriage confers the status of inheriting male on the daughter and adopts an in-marrying male as the symbolic "wife". However, in practice, the in-marrying husband typically carries out male duties in village social and ritual activities ^12^.

Young women participants were under-represented both sites. In Tumbakasa's (rural) case, according to the Banjar head, most of the young people have migrated to cities around Bali for school or work. In *Banjar* Biaung's profile in 2020, married women in the range of 20–29 years represented only 8.77% of the population. The age distribution of the twelve women, who were chosen as participants was beyond the control of the researchers because the RNG application randomly selected them. This was a limitation of the simple random sampling method for selecting small samples in this study. At the same time, the higher age range of respondents had the advantage of indicating long-term fertility outcomes as well as attitudes toward fertility for a substantial proportion of respondents.

## Results

The form of marriage in this patrilineal culture, which formally preferences the male line, critically influences Balinese women's legal status because it determines rights and responsibilities in the family and the broader customary community ( *banjar/desa adat*) ^12^. Most Balinese women participants feel the strain from the system arising from patrilineal marriage, especially the need for an heir, which tends to translate male precedence into son preference. The following discussion covers two themes. The first section includes rural and urban Balinese participants' stories of their experiences in the standard *purusa* marriage system and the importance of having a male heir. The second section shares their experiences and opinions about normative female exogamy, son preference, and the ubiquitous but less valued *sentana* option by which a daughter is symbolically taken as heir to continue the family line, with her spouse marrying into the household.

### a. Stories of *purusa* marriage

*Purusa* marriage refers to the male line through which the responsibility to maintain and carry out ritual ceremonies at ancestral shrines ( *sanggah*) and customary obligations ( *ayahan*) to the banjar community and *adat* village are inherited. It defines the structural power of the male position in the Balinese patrilineal family system, especially concerning inheritance and social/ritual obligations ^g^ ^32^. Seven of twelve Balinese women from the two study cohorts shared their experiences of their position in the household and society in this patrilineal system. These stories reflect various experiences related to this standard marriage pattern, with implications for family planning, fertility decisions, and women's role in society.

***a.1 Urban women's purusa marriage and fertility experiences.*** During this research, *purusa* marriage was prominent in Denpasar (the capital of Bali Province). Five of six urban women respondents had a *purusa* marriage. The following quotes from the in-depth interviews delved into their marriage experiences and implications for fertility decisions.

**Kota01 (46 years old, urban Balinese, a small retailer, mother of two daughters and a son)**
Indeed, in Balinese society, [women] should have a son. If they don't have a son, they will always try, and it can mean having five kids. Commonly, they will stop if they already have too many kids. Maybe they could ask for a son. *Nyentana,* where the man marries into the woman's family... There is a stigma of being a *sentana.* So, many men don't want to do *sentana*, even if they have economic limitations [in their household of birth].
I agree with the national program of two-children. I do not have two, but three because Balinese must have a son for the family line. A girl will get married and leave; a son will stay at home. *Purusa* is the [customary] *adat* system of succession. I have three children, and the last is a boy. The first two children are girls.
... after the second child, I got pregnant with a boy. Then, I stopped [having more children] and used contraception. Having three children was not my intention. Maybe if I had a boy and a girl, I would have stopped. My husband wants one more because he likes babies. *Aduh* [voice of distress] no more! It's enough. Taking care of a baby means I cannot go to work, or to socialize. I cannot go to the *banjar*, only stay at home. My husband accepts this [the decision not to have more children], and I never take out my IUD. It's my choice [to use contraception]. I won't have more kids. I have two girls and a boy - Complete!

In her account, Kota01 described common Balinese fertility attitudes and values revolving around the intense social pressure under the patrilineal system for having a son. The couple's fertility decisions were strongly influenced, on the one hand, by the pressure to have a male heir and, on the other, by her desire to limit the number of children through family planning. After having a son, the participant asserted her autonomy and, despite her husband's preference for another child, chose to stop having more children and to use contraception. This story underscores the complex interplay between cultural norms, personal choices, and societal expectations in shaping family dynamics and reproductive health decisions.

**Kota03 (36 years old, urban Balinese, office worker, mother of a daughter and two sons).**
I have three children…. Actually, I wanted two kids, but the Balinese have *sanggah gede* [core family temples with major ceremonies]. Only my parents [in-law], husband, and brother-in-law [are responsible]. So, we needed to have heirs. Three kids are enough for me because of the economic burden first and then *adat*…All of it needs money….
I had stress when I got pregnant with my first child. Moreover, my husband [always said] "boy" "boy". Not other people, my own husband. He asked, "Is it a boy, doc?" Then the doctor lectured my husband, "You should be grateful for having a kid when others can't." After that, my husband said, "If it is a girl, we can try again." Yes, a boy is expected because he will stay at home. He will be responsible for the house. …..
It is not the mother's fault [for only having daughters]. Yes, women are always blamed... I have a friend who has all-female siblings. What else can they do?... All get married, they leave [the parents]. Sometimes, they will come to visit. They did not marry *sentana*. Only one daughter stays with the parent until they pass away. After both parents have died, then [the house] will be handed over to the extended family.
A woman should be strong to be able to make decisions. [Wishing for] equality, most women take on more jobs, at home, at the office, for *adat,* but still have to respect their husbands. He is the decision-maker, but there should be teamwork. ….. I think it is important to nourish that concept in a family environment. I want my boys to be able to prepare offerings ( *nanding canang*), one of the women's customary roles. I teach them, even though they are boys. Sometimes they refuse because it is the woman's job. I like it when men can help with the "women's job". Because women are prohibited from making offerings *(banten*) during their menstrual periods, so the boys can help.

Kota03's statement also revealed the pervasive importance of the male line in Balinese culture and the intense pressure to produce a male heir from her husband and in-laws, rationalized by ancestral inheritance and the male head of household norms. Women often face blame for not having male children, reflecting deep-seated gender biases. This problem can lead to feelings of inadequacy and societal pressure, despite the biological reality that gender determination is not within their control. In this story, the participant expressed resentment of the patrilineal burden and pushed to modify aspects of the customary division of labor in socializing her children. Moreover, teaching boys to participate in traditionally female roles helps challenge gender norms and promotes a more balanced distribution of household responsibilities.

**Kota04 (41 years old, urban Balinese, civil servant, mother of a son).**
Thank God, I have a boy. I can be more relaxed. Balinese society prefers a son. Even if there is only one child, I still feel more relieved than if I had a girl. However, both are equal. It's still uncertain that boys can take good care of their parents. Still [a boy is always desired] because he is the family successor. We cannot move the *sanggah* [house temple]. The daughter should leave after marriage. In some cases, women will ask their boyfriends to marry *sentana*. They will change their positions so that the woman is the head of the family...
... I want to help [by working] because I see my husband only has a moderate economic status, and I have skills to work. My husband also supported me, even after I had a child. There was a time when I didn't have anyone to care for my little one. My sister-in-law also said, "Don't quit. What can you use to buy milk? Everything is expensive. We will work out who can take care of him". Sometimes, I took him to my office, which was all in the past. No one [protested] because my co-workers are all women in the same boat.

Like Kota03, Kota04 also viewed economic difficulty as a strong consideration in reproductive decisions. She argues that a son or daughter has equivalent value. Nonetheless, the community still privileges a boy for family continuity while recognizing *sentana* marriage as a solution for those with no male heir. Somewhat compensating for the inequality of the normative Balinese marriage pattern is the support from her husband, extended family, and colleagues in recognizing this triple burden, especially in resolving the demands of professional and parenting roles. She emphasizes the importance of support, flexibility, and shared responsibilities in achieving a balanced and fulfilling family life.

**Kota05 (46 years old, urban Balinese, an assistant at a nursing home, mother of two daughters and a son).**
Previously, I only wanted to have two children because, under Suharto [New Order era], KB [family planning acronym] was two. But at that time, I didn't have a son. Then at 38–39 years old, I had a boy. .... Absolutely, I would feel distressed if I didn't have a son. The pressure was from myself, as well as from my family. My husband has a nephew, but having my own son is different. Every day, I prayed for a son, so my family could have heirs for our legacy– a cultural legacy. If I were still younger, I would want more [children]. In the Balinese Hindu religion, having more than one boy can lighten up [by sharing] their obligation to take care of their parents, especially for *ngaben* [Balinese cremation] and the series of post-creation ceremonies. It is too hard for only one son.
... I am grateful. Although there is a lot of preparation for *adat* ceremonies, I don't feel tired. It is compulsory to pray.... but there is no burden. If we enjoy the process, we will never feel tired... I can manage it all... I would be more stressed if I didn't have a son.
When we have a load of *adat* work to do, the bits of help from others can make my day. For example, my husband will help me when I have a lot of *jaritan janur* [coconut leaf offerings to be prepared for *adat* ceremonies]. I feel happy. So, Balinese women don't feel alone.

Kota05 also expressed the social pressures that led her to have a third child. She was well aware of Indonesia's two-child policy campaign for economic well-being. However, her need to bear a son was absolute. The desire for a son could lead to emotional distress and a strong sense of relief and fulfilment when a son is born. This highlights the deep emotional impact of gender expectations on parents. Her fertility preferences were not based on an ideal number of children, but rather on the importance of a male child to support household ritual obligations. In her case, the cultural pressures placed on Balinese women produced a double-sided consciousness of the heavy load of *adat* tradition on the one hand and the contentment that came from the collective satisfaction of those obligations.

**Kota06 (37 years old, urban Balinese, a school administrator, mother of three daughters)**
From the government's point of view, the KB program is promoted for population control, but I never use KB [contraception]. So, I do not understand much about it. ... The wish [to have a son] is still there because of Balinese *adat,* [we must preserve] tradition into the future. I wish [to have a son], but back to the economic problem [of having more children]. So I am in the middle of yes or no.
There was no pressure from family because this era was not as traditional as it used to be, especially now that people can think ahead. So, we are not too [fanatical]... *Astungkara* [thank God], so far, in-laws or parents have never asked me to have a son or try again.
Yes, my husband still expresses his worries about the future if we only have daughters, "Who will take care of us?" Ah, now the era is different... *Nyentana.* Asking for the boy to come to the girl's home. With *sentana*, the system is better. If the boys are reticent, we will personally invite them to stay with us. ... but they will still do the banjar *adat* and civil obligation at the boy's own family's house. ... a double *banjar* obligation [ *pada gelahang*] ^h^.

Family planning and contraceptive decisions are heavily influenced by cultural and religious beliefs. Kota06 expressed considerable ambivalence in describing her fertility decisions. She did not use contraceptives even after having three children due to the high value of a son in the Balinese community. Aside from *adat* obligations, support in old age is a crucial consideration arising from patrilineal arrangements. While *sentana* marriage for one of their daughters would resolve this dilemma, there is a normative patrilineal model that weighs against an equal valuation of the *sentana* option. She also considered a new form of marriage *pada gelahang* that has evolved as a potential means to relieve pressures on both families.

The stories above show that even urban women who have careers outside the home felt pressure to have sons, and at least four out of five who have *purusa* marriages had an additional child to ensure there is an inheriting son to fulfill their task as Balinese wives. Three participants said they did so explicitly to satisfy patrilineal inheritance norms. It is undeniable in the Balinese context, that normative social expectations of women's roles shape and influence their fertility choices and reproductive agency. In this social construction, "women's destiny" was commonly idealized as a measure of the quality of womanhood. This is the case in Bali as well as across Indonesia ^33^.

***a.2 Rural women, family planning and purusa marriage.*** The conventional description of a woman's place in Balinese social structure is expressed in the following rural woman's perspective on marriage:

**Desa01 (47 years old, Balinese villager, a homemaker and casual farmer, mother of two daughters).**
I would happily have more kids if they have a [guaranteed] future. If they don't have it, I won't. I am not going to be forced to have more children... two are enough ... I cannot provide enough food for many kids. If other people have more (money), they will have four ^i^ For me, two are enough. It is okay... more importantly, I can take care of my kids within my limitations….
At 18 years old, she [one of the participants' two daughters] got married by *ngidih sentana* [seeking *sentana]*. *Ngidih sentana* is difficult. I don't know about [how she did it]. After four days, they got married. ..... I feel grateful to have gotten a *sentana* even if I don't have [assets]. I am really grateful. I have two grandchildren [a boy and a girl]. Safe. It was hard to find *sentana* because fewer [men] would marry outside... [Even] if [the parents] have three sons, they will prefer to stay at home. It was hard. He [the son-in-law] is from Kintamani, Bangli. He consented to a *sentana* marriage because he had five siblings, three of them were boys who had only a single cramped house.

As a casual farmer and homemaker, Desa01 explained that economic limitations outweigh her fertility preference to have more children. She stopped after two pregnancies, even though she had no son. Not only did economic constraints influence her fertility decisions, but analogous economic difficulty by her account was a driving factor of her son-in-law's agreement to a " *sentana*" marriage with her daughter. Despite economic limitations and the challenges of finding a *sentana*, there was a sense of gratitude and adaptation. The story reflects the ability to find solutions within cultural constraints and the importance of being grateful for what one has.

**Desa04 (41 years old, Balinese villager, homemaker and casual farmer, mother of a daughter and a son).**
It is better to have two [children]. I already have a boy and a girl. I am grateful. I wanted that, and I got it. I use the IUD. It fits...... and is safe. No [side effects or complaints]. I feel comfortable.
The pressure to have a son is still intense in my community. It will always be problematic. Having two daughters is a problem. Having *sentana* is still a problem. Then, telling the daughter to make a lot of boyfriends that could be a [marriage] candidate always becomes a problem. After she grows up, the daughter still gets blamed.... the parents complain, and the daughters are more upset. It's a pity.
Indeed, [Balinese women] obey their husbands and are submissive. I don't want my husband to follow me. I don't want to. I was born and aware of my situation as a woman.
No, I am a follower. My in-law and my husband had better make the decisions. I don't want to get involved. I think I am not able, incompetent. It is better that I obey.
Because I stay with my husband's family, he carries more weight. It would be different when the husband lives with the wife's family. Also, I no longer have rights at my family's house because I got married outside. I only give them a visit…. I am a woman.
When we have many *adat* ceremonies *....* sometimes we complain. Even though it is a Balinese routine, I usually share my weariness with my friends. When we share and talk in the *banjar*, I feel more relieved.

Desa 04 shared her experiences as a rural Balinese woman who left her natal family for the normative *purusa* marriage. She eventually exercised fertility autonomy in her decision to use contraception after fulfilling her obligation as a wife to bear a male heir for her husband's family. Traditional gender roles emphasized obedience and submission of women to their husbands and in-laws. This reflects a deeply ingrained cultural norm where women often defer decision-making to male family members. Her story portrayed a typical rural Balinese woman as a victim of the patrilineal system in society, normative values she has primarily internalized. Nonetheless, the women's sphere also offers a support network that accompanies the intense social interaction in ritual and community work. Exchanging stories in the *banjar* made her realize that she was not alone.

Differing from their urban counterparts, the rural Balinese women’s accounts provide more balance between cultural expectations (such as having heirs and maintaining family traditions) and practical realities (such as economic limitations and the ability to care for children). In their narratives, Balinese women in *purusa* marriage share problematic living arrangements and rights. The living arrangement, where a woman stays with her husband’s family, places additional responsibilities on the husband and limits the woman’s rights in her own family’s house. This highlights the gendered nature of family dynamics and inheritance. The stories illustrate the ongoing negotiation between adhering to cultural norms and making personal choices that align with personal comfort and well-being. Women navigate these expectations while finding ways to assert their agency within the constraints of their cultural context.

### b. Stories about *sentana* marriage

In Balinese society, typically, male descendants have rights to family inheritance. In an alternative marriage system partially parallel to female exogamy, men who marry *sentana* lose these rights. Men no longer have formal rights or responsibilities after they leave their natal family ^32^. Moreover, *sentana* marriage is a step taken to maintain the family line even though the family only has daughters. This marriage aligns with the national family planning program focusing on small, happy, and prosperous families benefiting from Balinese society's development ^12, 36^.

The Balinese community's acceptance of this marriage form varies and depends on each region's *adat*. Tabanan and Gianyar regions accept and have practised *sentana* marriage from generation to generation ^12^. Other areas of Bali province, such as Jembrana, Karangasem, and Klungkung, have not recognized *sentana* marriage ^12^. This uxorilocal marriage has a weak legal status and misses the three legal components of law: legal structure, legal substance, and legal sanctions ^12, 18, 37^. S *entana* marriage is an *adat* (customary) law practice, not a rule of Hinduism ^12^. This marriage depends on the local provision of *awig-awig* or Balinese customary law, which may differ from one *adat* village to another in Bali ^18^. Five women were in a *sentana* marriage relationship among the twelve rural and urban interview participants. However, interestingly only one of these was an urban participant, which might have been expected because *sentana* marriage is not common in the Denpasar community ^12^.

**Kota02 (45 years old, urban Balinese, *sentana marriage*, homemaker and online retailer, mother of two daughters).**
In Balinese culture, women marry outside. It is an obligation. Because my brother passed away, I had to stay at home. I asked [my husband] to stay with me because of that situation. Yes, *nyentana...* I cannot marry outside because there are no heirs. I have cousins, but the one who has rights here is me. My husband agreed to do it [ *sentana*]. He bears a heavier burden because he also had to change his faith ^j^. His family agreed. It is also what he wants. I am not pushing him. If I force him, I'm afraid it will be a problem in the future. Commonly, after *sentana* and having children, the husband leaves.
If I had two sons, two would be enough. Because I have two girls, I want one more child. If it is not my luck, it won't matter. Enough. Girls are the same [as boys]. [I'm] still grateful. One of them can stay at home, like me. I told my husband we would always be grateful if we only had two girls.

In the experience of Kota02 the pressure did not stop after she found a partner willing to marry *sentana* but continued after her two pregnancies. She felt that she needed to have a son, or one of her daughters would repeat her experience because of the strength of the patrilineal system in the Balinese community. In her *sentana* marriage, Kota02 recalled that her husband also experienced pressures due to their marriage, which points to other perspectives on the experience of *sentana* for men who can also be 'victims' of cultural values and socio-economic structures.

**Desa02 (47 years old, Balinese villager, *sentana marriage*, a homemaker, mother of two sons).**
It is easier for a man to find a woman but harder for a woman to ask a man to stay at her home. Balinese men have a different status. ... A woman will not be[come] a man [through *sentana*]. The head of the household is still the husband, not me. The difference is that the inheritance is the woman's treasure.
I am one of two sisters. One left the house. I stayed, and sought *sentana.* I lived and was born here. I found a husband [a neighbor]. I had [two] sons. A neighbor took one for " *sentana*". ... No one leaves their parents here. ... Together, they care for their parents... It is not a big deal for me [because] my [other] son, his wife, and grandchildren often visit.

Desa02 reflected on her fertility decisions as a rural woman and homemaker in the context of her own *sentana* marriage and that of one of her sons. She was grateful that she has two sons and that the one who lives with his wife is close enough to visit his mother often. A mitigating factor in both types of marriage is the strong traditional preference for in- *banjar* (hamlet) marriage, which means that close relationships are usually maintained regardless of the marriage pattern. Interestingly, this story also emphasizes the importance of family responsibilities and care for parents. Even when children marry and move out, there is a strong sense of duty to care for their parents, often resulting in close-knit and interconnected family structures.

**Desa03 (43 years old, Balinese villager, *sentana marriage*, a homemaker and craftswoman, mother of two daughters).**
I have two girls. No, no, no. I don't want to have more. Two are enough. It is easier. The issue is economic. I only have cramped land. Where will they stay? Rich people have more children. I accept my fate.
I was *ngidih* [proposing *sentana*] because I was the one and only heir of my family. It was hard [to persuade the prospective husband to *nyentana*]. Today's era is even more difficult. In the past, my parents took care of it. They did not introduce him to me, but our marriage was an arrangement. Now, children won't do that. She will look for [a husband] by herself. I was stupid. I can't do things right... more important to have a descendant.
I want to have a son…. but what if I have more kids, and it is a girl... I lay my faith in God. [one of the participant's daughters is prepared to marry *sentana*].
My husband is still the head of the family. Even though he is from the outside, he is still …. a man. Always, because he is the man and I am only a woman. As a woman, I don't dare to disturb my husband, asking for this and that. I don't dare. Let him be. Yes, my husband is still 'number one'.

The pressure on Balinese women does not stop after finding a *sentana* candidate and marriage. Male heirs are still critical for *adat* continuity in the next generation. Desa03 experienced a difficult and rocky journey as a daughter with no brother, the wife of *sentana* husband, and a mother with no son. Social pressures were multiplied by economic limitations as a rural homemaker and craftswoman. For her, the economic factor ultimately outweighed any advantage in producing a male heir. Even though the desire to have a son persists, there is also an acceptance of whatever outcome occurs, with faith playing a role in accepting the gender of future children. Desa03 accepted their economic limitations and the cultural expectations placed upon them, finding ways to navigate these challenges within their means.

**Desa05 (31 years old, Balinese villager, *sentana marriage*, an honorary teacher, mother of two daughters).**
Two children would be enough if I had a son and a daughter. Sadly, I have two girls. Yes, later [maybe another baby]. The pressure is actually from me. I really want [a son]. I'll be grateful if I have one, but it'll be okay if I don't. More importantly, I have children rather than nothing...
Balinese men prefer to stay at their own home, so asking them to stay at a women's place will be hard. Rarely will the men obey. ... Maybe, it's more comfortable to stay. Moreover, finding several brothers who will remain at their family's home is essential. That's the difficulty.
*Aduh,* Balinese have a lot [ *adat* activities]. I have been performing *adat* work ( *ngayah*— Balinese mutual cooperation) for one week at the temple *.* Then, two days later, I will have a ceremony at my house. I am still fortunate to have a mother and aunt who always stay with me and can help [thanks to her *sentana* marriage], so I can go to work. In this *banjar*, mutual help among neighbors is a tradition.

In concert with Desa03, Desa05 shared her story on population policy versus patrilineal pressure. She also experienced Balinese women's triple burden, which is productive (working as a teacher), reproductive (bearing male heirs), and customary (ritual *adat* activities). However, the participant gained support from other women and neighbors to alleviate her triple burden as a rural Balinese woman. This story illustrates the resilience and adaptability of individuals in navigating cultural expectations and personal circumstances. Desa05 found ways to fulfil cultural obligations while also managing work and family life.

**Desa06 (40 years old, Balinese villager, *sentana marriage*, a small retailer, mother of three sons).**
Men are expected to be the heirs of the family. It is the principle. If the first baby is a boy, the second will be easier; whether a boy or girl doesn't matter. If the first child is a girl, the second will be more difficult. Looking for *sentana* is harder. Yes, it was not easy when I was looking for [ *sentana*] here [neighborhood]. I had to go looking far afield. .... Men rarely want to look for *sentana.* Men think it is better to stay at home. Doing *sentana*, men live in someone else's family...
I was *ngidih* (looking for) a *sentana*... I have two siblings, all girls. Now, I have three boys. ... I should obey my husband to keep him calm—making him comfortable, following him. I should control my speech so that he won't get offended. I will comply with my husband's decisions and not cause him distress; make him feel at home. .... I am indeed the heir, but he is responsible for the kids' tuition fees. As a woman, how much do I earn?
The husband's role is more important than mine as a woman. He is the leading actor and takes responsibility for everything, including *adat*. I feel sorry for him when there are more demands, like the children's tuition fees. He gets a burden, but I cannot help. Sometimes, I feel sorry.
Suppose other families ask for a son [because I have three]. If my son wants to, I will give him to them. Yes, I experienced the difficulty of looking for a *sentana.* [But] I also should be ready for all my kids if they choose to stay at home after marriage.

Even though she sought *sentana* and had the right to her family inheritance, Desa06 still felt inferior to her husband. She felt the need to compensate for his agreement to enter a *sentana* marriage. Desa06's story reflects a process of learning from the " *ngidih sentana"* marriage experience, which contributes to her personal openness to a son following this path out of sympathy for both the needs of other women in this position as well as the personal marriage choices of her children.

From the *sentana* marriage stories above, we can learn that the principle of cultural expectation for men to be the heirs of the family influences fertility decisions even where “ *ngidih sentana*” is prevalent. Patriarchal and patrilineal systems underscore the husband’s authority and the wife’s compliance. This dynamic often leads the wife to adhere to her husband’s decisions and strive for household harmony, reflecting deeply entrenched cultural norms regarding gender and authority. The readiness to conform to cultural expectations, such as transferring a son to another family, if necessary, demonstrates flexibility in navigating traditional practices. This approach reflects a balance between upholding cultural norms and adapting to individual circumstances.

## Discussion/Conclusion

The strong patriarchal norm in Indonesia contributes to women's social problems ^38^, especially Balinese-Hindu women. Balinese Hinduism profoundly influences cultural norms and practices across Bali, embedding religious ceremonies and rituals in daily life. Urban and rural variations in fertility preference highlighted the patriarchal system which remains at the center of Balinese society's social structure through patrilocal and patrilineal religious and customary traditions ^24^. The system pressures married Balinese-Hindu women to have children, especially a son as an heir, and to continue the husband's family's lineage ^24^. The emphasis on having a son causes some women to add to the number of children they have with the hope that the next child will be a boy ^39^. The interviews with rural and urban women revealed that most participants experience the pressure to have a son in their marriage as a burden.

The Balinese patrilineal inheritance system places the male at the center of the social structure ^32^. Male descendants are considered the main actors who carry the inheritance, name, and responsibility for the family, making them highly valued in the community. Balinese women participants revealed their fertility attitudes in the context of structural pressures favoring patrilineal ( *purusa*) marriage and masculinist values. The variations in women's responses reported above are based on their realistic recognition of social norms and personal life circumstances. Son precedence, if not necessarily preference, is an outcome of those structures and is mainly responsible for the fertility choices and constraints on their perceived options ^21^. Several respondents in both *purusa* and *sentana* marriage types decided to use contraception after they gave their husbands a son. Most others did not use contraception because they have no son yet. This finding aligns with other studies in Bangladesh where contraceptive use among women with only daughters was lower than women with sons(s) because of the influence of the patrilineal system. Consequently, son(s) preference became a reason to have a larger family size when they only had daughters ^40^.

Balinese-Hindus believe the ancestral spirit is released from the abyss through the grandson's role ^41^. In Balinese, getting pregnant and giving birth to a child is more than a biological matter. It is connected to culture and religion ^42^. As written in *Rgveda* X.85.42: " *Ihahiva stam ma vi yaustam, visvam ayur vyasnutam Kridantau Putrair naptrbhih, modamanau sve grhe*", meaning: "Yes husband and wife may you stay here and never be separated. Hopefully you both achieve full life happiness. Hopefully you play with your son and your grandsons, and live in this house happily" ^43^. The ancient literature confirms the strain on the family member to maintain lineage continuity. In a *sentana* marriage, the burden of having a son continues into subsequent women's generations in the family, from a mother to her daughter. It is illustrated by Desa01, who chose not to add another child to the family for serious economic reasons when she had no son. Her decision passed an extra obligation upon her daughter to find a man willing to accept “ *sentana*” marriage and give birth to a grandson as an heir to give her family a sense of security.

Despite strong cultural pressures and preferences for male heirs, several rural Balinese women assert their autonomy by making informed reproductive health choices, such as using contraception ^44^. Another study in remote Bali explained that younger women with fewer children, recent births, and regular health service access were more likely to desire more children ^45^. The study also mentioned that Balinese women who desire more children were likely to use contraception, but this recent study indicated that the use of contraception was connected with economic liability rather than age group or health access. Prioritizing family well-being and economic stability over cultural norms marks a shift in gender dynamics. Support from husbands can be crucial, highlighting the importance of male involvement in the family dynamic. This collaboration promotes shared decision-making and challenges patriarchal norms, enhancing women’s autonomy and gender equality in reproductive health decisions.

Economic pressure is a structural factor that greatly outweighs son preference in the fertility decisions of these women, particularly in rural areas. This is particularly evident in the rural Balinese women's perspective on *sentana* marriage. The result is in line with a study result in other rural areas in Bali that Balinese women's fertility aspirations are not simply left to God or fate but determined in concert with their husband (and extended family), considering the costs and benefits of child-bearing and childrearing ^21^. The assumption that men's status after *sentana* marriage as *predana* or "woman" ^12, 32, 37^, makes their position inferior is not automatic. As portrayed by some respondents' stories, the husband was still the man of the family, and the wife still felt obliged to obey her husband. In one case, a respondent described feeling the need to serve her husband courteously to honor his "sacrifice" as " *sentana nyeburin*" ^k^. That said, some accounts also described supportive husbands and extended families.

The findings reveal the multiple roles of Balinese women as wives, mothers, and members of society. The results also show that marriage patterns and fertility decisions face significant cultural and economic drivers. *Sentana* marriage, considered a solution to the prescription for male inheritance, cannot guarantee Balinese women's equality. This marriage could be deemed a tool to prevent the loss of the family line and inheritance and ultimately serve to maintain patriliny. Challenges are raised not only by customary law but also by constraining economic factors.

Impressively, this study provides a narrative showing Balinese women as more culturally adaptive than men. First, women frequently juggle multiple roles within their families and communities, such as caregivers, homemakers, and workers, necessitating high adaptability to changing circumstances and diverse social expectations ^46^. Second, social norms often place women in positions requiring flexibility and responsiveness, especially during crises where they manage household needs and care for vulnerable family members ^46^. Additionally, women often have differential access to resources and information, driving them to develop adaptive strategies to navigate these limitations effectively ^47^. Studies show that women are perceived as more compassionate and sensitive, enhancing their ability to build and maintain social networks crucial for adapting to cultural and environmental changes. Historically, women have adapted to various societal changes and challenges, such as shifts in family structures and economic roles ^48^. Evolutionary biology also suggests that women have developed adaptive strategies to ensure the survival and well-being of their offspring ^49^. These factors collectively contribute to the perception and reality of women being more culturally adaptive than men globally.

While not generalizable beyond this small sample, this study, using in-depth interviews, indicates the complexity of internal and external forces affecting Balinese women's fertility decisions. Quantitative and qualitative studies are needed to provide broader perspectives and deeper understanding, especially regarding Balinese women's fertility agency in the context of culture, customary law, and development policies. Tailoring interventions to the specific needs of different regions makes them more effective. Support from husbands and family members is crucial for women to make their own reproductive choices. Engaging men in family planning can change traditional norms and support women’s autonomy. Providing accessible and culturally sensitive family planning services and education is essential ^51^. Policymakers must consider cultural, economic, and social factors when designing reproductive health policies. Supporting women’s rights and autonomy should be a priority. Continuous education and advocacy are needed to challenge and change entrenched cultural norms. Promoting gender equality and reproductive rights requires ongoing, multi-level efforts. A holistic, context-sensitive approach to reproductive health recognizes the diversity of influences on women’s decisions.

## Data Availability

The audio files will not be shared because the interviews may contain sensitive data about personal life and community. However, there is provision for some de-identified transcripts to be provided on request to ensure that use is limited to genuine researchers and to protect respondents’ privacy and the local community. An application from an established researcher to obtain redacted transcripts would require review by the HREC Committee at Murdoch University. A written request outlining 1) the name and status of the researcher, 2) institutional affiliation, and 3) purpose for which the data is sought, would be required of the applicant. Figshare: Stories of women's marriage and fertility experiences :Qualitative research on urban and rural cases in Bali. https://doi.org/10.6084/m9.figshare.23674401.v1 ^52^. This project contains the following extended data: Approval Letter _Balinese story.pdf Oral Information and Consent Form-33512093.pdf Stories of women.docx (interview guide) Data are available under the terms of the Creative Commons Attribution 4.0 International license (CC-BY 4.0).
