# Supplementary material for: Stories of women's marriage and fertility experiences: Qualitative research on urban and rural cases in Bali, Indonesia
Source: Gates Open Res. 2024 Oct 14;7:124. Originally published 2023 Oct 11. [Version 2] doi: 10.12688/gatesopenres.14781.2 (PMC11480341; doi:10.12688/gatesopenres.14781.2)
Supplement: Peer review history

# Reviewer report: Reviewer response for version 2

**DOI:** 10.21956/gatesopenres.17689.r38260

**Published:** 2024-10-15

**Authors:** Premchand Dommaraju

**Affiliations:** 1 Nanyang Technological University, Nanyang, Singapore

I have no further comments.

Is the work clearly and accurately presented and does it cite the current literature?

Partly

If applicable, is the statistical analysis and its interpretation appropriate?

Not applicable

Are all the source data underlying the results available to ensure full reproducibility?

Partly

Is the study design appropriate and is the work technically sound?

Yes

Are the conclusions drawn adequately supported by the results?

Partly

Are sufficient details of methods and analysis provided to allow replication by others?

Partly

Reviewer Expertise:

Marriage, families, and households in Southeast and South Asia. Demography.

I confirm that I have read this submission and believe that I have an appropriate level of expertise to confirm that it is of an acceptable scientific standard.

# Reviewer report: Reviewer response for version 1

**DOI:** 10.21956/gatesopenres.16107.r37575

**Published:** 2024-08-23

**Authors:** Made Diah Lestari

**Affiliations:** 1 Udayana University, Bali, Indonesia

1. Statement: "triple burden", can you explain more what the authors mean by "burden" instead of triple roles? Can you demonstrate the burden in detail?

 2. Statement: " Indonesian government policies on family planning and local concern with cultural preservation significantly influence their marriage and fertility decisions" - how do these two situations influence women's fertility decisions? 

 3. Are there any specific demographic features of these two locations (e.g., number of males, number of females, fertility rate) that are reasons for the author to choose these two locations to recruit the research participants?

 4. "Researchers used data triangulation with in-depth interviews, document analysis, and observation to enhance the validity of the research" We usually use 'credibility' instead of 'validity' in qualitative research. 

 5. How did you integrate document analysis (e.g., local government regulations, documents on Balinese customary law, and publications) into your primary data?

 6. Can you explain more about the method, what is a technique used to analyze the primary data? Can you explain more about the process? 

 7. Here is just my suggestion for your finding:

 a. Please do review Braun and Clarke's checklist.

https://bmjopen.bmj.com/content/bmjopen/11/7/e054368/DC3/embed/inline-supplementary-material-3.pdf?download=true

 to improve your analysis and the way you present your findings.

 b. Structure of the findings:

 - Fertility experiences (kota and desa)

 - Patrilineal/purusa marriage (kota and desa)

 - Sentana marriage.

Is the work clearly and accurately presented and does it cite the current literature?

Yes

If applicable, is the statistical analysis and its interpretation appropriate?

Partly

Are all the source data underlying the results available to ensure full reproducibility?

Yes

Is the study design appropriate and is the work technically sound?

Partly

Are the conclusions drawn adequately supported by the results?

Partly

Are sufficient details of methods and analysis provided to allow replication by others?

Partly

Reviewer Expertise:

Ageing studies, local knowledge, critical psychology, and culture

I confirm that I have read this submission and believe that I have an appropriate level of expertise to confirm that it is of an acceptable scientific standard, however I have significant reservations, as outlined above.

# Response

**Published:** 2024-10-02

**Authors:** Anastasia Septya Titisari

**Affiliations:** Research Center for Population, National Research and Innovation Agency Republic of Indonesia, Central Jakarta, Jakarta, Indonesia

Dear Dr. Lestari,

 Thank you for your thorough review and valuable feedback on our manuscript. We appreciate your insights and have addressed your comments as follows:

1. **Clarification on “Triple Burden”**: We have elaborated on the term “burden” to distinguish it from “triple roles”.
2. **Influence of Government Policies and Cultural Preservation**: We have expanded the discussion on how Indonesian government policies on family planning and cultural preservation impact women’s fertility decisions.
3. **Demographic Features of Locations**: We have included demographic details of the two locations, explaining the rationale behind their selection.
4. **Terminology in Qualitative Research**: We have revised the terminology to use ‘credibility’ instead of ‘validity’ in the context of qualitative research.
5. **Integration of Document Analysis**: We have clarified how document analysis was integrated with primary data, detailing the process and its contribution to our findings.
6. **Data Analysis Method**: We have provided a more detailed explanation of the data analysis techniques.
7. **Structure and Presentation of Findings**: We have reviewed Braun and Clarke’s checklist and restructured our findings to improve clarity and presentation.

We hope these revisions address your concerns and enhance the quality of our manuscript. Thank you once again for your constructive feedback.

# Reviewer report: Reviewer response for version 1

**DOI:** 10.21956/gatesopenres.16107.r37578

**Published:** 2024-08-10

**Authors:** Nina Nurmila

**Affiliations:** 1 Universitas Islam Internasional Indonesia, Depok, Indonesia

The article provides insightful stories of gender inequality experienced by Balinese women due to patriarchal and patrilineal system. However, there are technical and substantive revision to improve the article:

 1. There are too many keywords, please choose up to five words which are distinctive about this article;

 2. This article does not have literature review to locate how this research fill the existing gap of knowledge

 3. In other journal articles and book, footnote number usually puts after punctuation mark, not before

 4. There is inconsistency of referencing style between footnote and intext reference such as (Adnyani 2017) in the Introduction.

 5. Methodology: (1) Triangulation is for the validity of DATA, not research; not "participant observation" but "researcher's observation on/over participants".

 6. There is lack of data analysis in the Discussion. The writers can use the existing literature to see the finding of this research. For example, what is missing is that even though some of the Balinese women felt being subordinated in Balinese patriarchal culture, there is agency of some of the research participants such as that one or several of them decide to use contraception to prevent from having more children regardless of cultural pressure of having son.

 7. The positive thing of this article which uses qualitative research is the direct quote from participants, even though it is better that authors do deeper analysis on these direct quote.

Is the work clearly and accurately presented and does it cite the current literature?

Partly

If applicable, is the statistical analysis and its interpretation appropriate?

Not applicable

Are all the source data underlying the results available to ensure full reproducibility?

Partly

Is the study design appropriate and is the work technically sound?

Yes

Are the conclusions drawn adequately supported by the results?

Partly

Are sufficient details of methods and analysis provided to allow replication by others?

Yes

Reviewer Expertise:

Gender and Islam in Indonesia

I confirm that I have read this submission and believe that I have an appropriate level of expertise to confirm that it is of an acceptable scientific standard, however I have significant reservations, as outlined above.

# Response

**Published:** 2024-10-02

**Authors:** Anastasia Septya Titisari

**Affiliations:** Research Center for Population, National Research and Innovation Agency Republic of Indonesia, Central Jakarta, Jakarta, Indonesia

Dear Nina Nurmila,

 We appreciate your constructive feedback and have made several revisions to address your concerns. Below are the key changes we have implemented:

1. **Keywords**: We have reduced the keywords to five distinctive terms that best represent our article.
2. **Literature Review**: We have added a comprehensive literature review better to situate our research within the existing body of knowledge.
3. **Referencing Style**: We have corrected the placement of footnote numbers and ensured consistency in referencing style throughout the manuscript.
4. **Methodology**: We clarified the use of triangulation for data validity and revised the term “participant observation” to "researcher’s observation on/over participants."
5. **Data Analysis**: We have enhanced the discussion section with a more profound analysis, incorporating existing literature to contextualize our findings. We also highlighted instances of agency among Balinese women, such as their use of contraception despite cultural pressures.
6. **Direct Quotes**: We retained direct quotes from participants but provided more in-depth analysis to capture the complexities of their experiences better.

We believe these revisions have strengthened our manuscript.

# Reviewer report: Reviewer response for version 1

**DOI:** 10.21956/gatesopenres.16107.r37580

**Published:** 2024-08-02

**Authors:** Premchand Dommaraju

**Affiliations:** 1 Nanyang Technological University, Nanyang, Singapore

The paper examines marriage and fertility decisions and the factors shaping those decisions among Balinese women. The paper gives voice to Balinese women's experience of marriage and fertility. The study is locally informed, highlighting the normative and cultural aspects and how they shape Balinese women's fertility and marriage experiences.

 The literature on son preference, including differential spacing and stopping behaviour, is extensive. The paper could benefit from reviewing and presenting the literature and situating the paper within the broader existing literature. This would allow the study to situate how the experiences of Balinese women differ from or are similar to those of women in other patriarchal and patrilocal systems in South Asia, East Asia, and Southeast Asia.

 While it is good that the manuscript cites sources not published in English for contextual information, it would be beneficial also to cite sources in English. This would aid readers who might not be proficient in the cited languages. Given the extensive body of English-language literature on Balinese social, cultural, and gender dynamics, this should be feasible. Additionally, though the use of Balinese terms enhances authenticity, moderating their frequency can improve readability without sacrificing accuracy.

 The methodology raises several concerns.

 It is unclear why the two locations were chosen, besides the fact that one was rural and the other urban. Are they from the same region within Bali? Are there significant differences in cultural, social, or economic aspects between these two sites?

 A clear justification for the sample size of 12 participants is needed. Was it based on data saturation or other considerations?

 Additionally, please provide the rationale for selecting women aged 30 to 49. With three-fourths of the women in the sample being older than 40, there are concerns about potential biases toward earlier fertility and marriage experiences. Discuss how the experiences of this cohort might differ from those of recent cohorts.

 The analytical approach needs to be clarified. The manuscript mentions in-depth interviews, document analysis, and observation, but it needs to be clarified how the other data sources were used besides the interviews.

 The paper mentions that the data were coded and interpreted, but no coding procedure is described. Please provide additional details on the coding procedure and analytical techniques used. Without a clear analytical approach, it is difficult to assess the validity of the results presented.

 The use of quotes is valuable for capturing women's voices. However, a more in-depth analysis beyond descriptive accounts is necessary to understand the complexities of marriage and fertility experiences. Also, the quotes are often too long and seem to amalgamate answers to different questions. Consider revising how the quotes are presented.

Is the work clearly and accurately presented and does it cite the current literature?

Partly

If applicable, is the statistical analysis and its interpretation appropriate?

Not applicable

Are all the source data underlying the results available to ensure full reproducibility?

Partly

Is the study design appropriate and is the work technically sound?

Yes

Are the conclusions drawn adequately supported by the results?

Partly

Are sufficient details of methods and analysis provided to allow replication by others?

Partly

Reviewer Expertise:

Marriage, families, and households in Southeast and South Asia. Demography.

I confirm that I have read this submission and believe that I have an appropriate level of expertise to confirm that it is of an acceptable scientific standard, however I have significant reservations, as outlined above.

# Response

**Published:** 2024-10-02

**Authors:** Anastasia Septya Titisari

**Affiliations:** Research Center for Population, National Research and Innovation Agency Republic of Indonesia, Central Jakarta, Jakarta, Indonesia

Dear Premchand Dommaraju,

 Thank you for your thorough and insightful review of our manuscript. Below, we address the key points you raised:

1. **Justification for Location Selection**:
  - We chose the two locations based on their distinct urban and rural characteristics within the same region of Bali. This allows us to explore the influence of different socioeconomic and cultural contexts on marriage and fertility decisions.
2. **Sample Size and Participant Age Range**:
  - The sample size of 12 participants was determined based on the minimum sample by Creswell & Poth (2018). We selected women aged 30 to 49 to capture a range of fertility and marriage experiences, acknowledging that older participants may reflect earlier experiences, as explained in the revised manuscript. 
3. **Analytical Approach**:
  - We provide a detailed description of our coding procedure and analytical techniques in the new version of the manuscript. This includes how we integrated data from in-depth interviews, document analysis, and observations to ensure a comprehensive analysis.
4. **Use of Quotes**:
  - We revise the presentation of quotes to enhance clarity and depth of analysis, ensuring they effectively illustrate the complexities of marriage and fertility experiences.
5. **Literature Review**:
  - We incorporate a review of relevant literature to situate our findings within the broader context of existing research on son preference and patriarchal systems in South, East, and Southeast Asia.

We are confident that these revisions will strengthen our manuscript and address your concerns. Thank you once again for your valuable feedback.
